# “Telepsychiatry: Lessons from the COVID-19 pandemic”

**DOI:** 10.1192/j.eurpsy.2021.320

**Published:** 2021-08-13

**Authors:** D. Mucic

**Affiliations:** Telepsychiatry, Little Prince Treatment Centre, copenhagen v, Denmark

**Keywords:** COVID19, telepsychiatry, regulations, licensure, malpractice

## Abstract

**Introduction:**

Under the umbrella of e-Mental Health (eMH), Telepsychiatry (TP) keeps its place as the oldest and best-documented application. Legislative issues, and the concerns related to the quality of care and patient safety, have kept TP from broader adoption. COVID19 pandemic seems to be a turning point for TP as well as for the eMH in general. The use of TP has exploded as many regulatory barriers to its use have been temporarily lowered during the COVID-19 pandemic. What has to be done to sustain this momentum?

**Objectives:**

-outline temporary changes in TP regulations made due to COVID19; -discuss which of these should be maintained, modified, or reversed; -suggest additional initiatives needed to facilitate patient and professional use of digital technology.

**Methods:**

Examination of the use of digital technology in the light of regulatory, legislative, and other changes and initiatives made due to COVID 19.

**Results:**

Among several policy changes, the most important is e.g. removal of the “originating site” rule so professionals can be paid for a remote appointment wherever the patient is, including in the patient’s home. Further, professionals were allowed to serve patients through everyday communication technologies such as FaceTime, WhatsApp, Viber, or Skype, all compromising patient/data safety.

**Conclusions:**

EPA is perfectly positioned to be the frontrunner for the required initiatives i.e. mandatory lectures related to eMH at medical educational institutions, launching of TP-competency training of mental health professionals, regulatory and statutory changes e.g. unified licensure regulations, etc that are crucial for modernizing mental health care delivery and preparation for future unprecedented events.

**Disclosure:**

No significant relationships.

